# The clinical course of Duchenne muscular dystrophy in the corticosteroid treatment era: a systematic literature review

**DOI:** 10.1186/s13023-021-01862-w

**Published:** 2021-05-22

**Authors:** Shelagh M. Szabo, Renna M. Salhany, Alison Deighton, Meagan Harwood, Jean Mah, Katherine L. Gooch

**Affiliations:** 1 Broadstreet HEOR, 201 – 343 Railway St, Vancouver, BC V6A 1A4 Canada; 2 Sarepta Therapeutics, 215 First St, Cambridge, MA 02142 USA; 3 Cumming School of Medicine, University of Calgary, Calgary, AB Canada

**Keywords:** Duchenne muscular dystrophy, DMD, Clinical course, Loss of ambulation, Systematic review

## Abstract

**Background:**

Duchenne muscular dystrophy (DMD) is a severe rare progressive inherited neuromuscular disorder, leading to loss of ambulation (LOA) and premature mortality. The standard of care for patients with DMD has been treatment with corticosteroids for the past decade; however a synthesis of contemporary data describing the clinical course of DMD is lacking. The objective was to summarize age at key clinical milestones (loss of ambulation, scoliosis, ventilation, cardiomyopathy, and mortality) in the corticosteroid-treatment-era.

**Methods:**

A systematic review was conducted using MEDLINE and EMBASE. The percentage experiencing key clinical milestones, and the mean or median age at those milestones, was synthesized from studies from North American populations, published between 2007 and 2018.

**Results:**

From 5637 abstracts, 29 studies were included. Estimates of the percentage experiencing key clinical milestones, and age at those milestones, showed heterogeneity. Up to 30% of patients lost ambulation by age 10 years, and up to 90% by 15 years of age. The mean age at scoliosis onset was approximately 14 years. Ventilatory support began from 15 to 18 years, and up to half of patients required ventilation by 20 years of age. Registry-based estimates suggest that 70% had evidence of cardiomyopathy by 15 years and almost all by 20 years of age. Finally, mortality rates up to 16% by age 20 years were reported; among those surviving to adulthood mortality was up to 60% by age 30 years.

**Conclusions:**

Contemporary natural history studies from North America report that LOA on average occurs in the early teens, need for ventilation and cardiomyopathy in the late teens, and death in the third or fourth decade of life. Variability in rates may be due to differences in study design, treatment with corticosteroids or other disease-modifying agents, variations in clinical practices, and dystrophin mutations. Despite challenges in synthesizing estimates, these findings help characterize disease progression among contemporary North American DMD patients.

**Supplementary Information:**

The online version contains supplementary material available at 10.1186/s13023-021-01862-w.

## Background

Duchenne muscular dystrophy (DMD) is a rare, progressive, life-limiting neuromuscular disorder [1] occurring in 15.9 to 19.5 per 100,000 live male births [2–4]. It is caused by mutations in the dystrophin gene [2, 5]; lack of dystrophin compromises muscle structure and integrity, leading to progressive muscular degeneration [6, 7]. Patients with DMD are typically identified in early childhood with symptoms including delays in motor milestones and frequent falls [8]. Over time, these patients experience progressive functional impairments leading to loss of ambulation (LOA), pulmonary insufficiency, cardiomyopathy, and early mortality [2, 5, 9].

Although there is presently no cure for DMD, advancements to the standard of care, including the introduction of systemic corticosteroids in the 1990s, have helped slow disease progression and improve survival [10–12]. However, the impact of these changes in standard of care across the full range of clinically-relevant disease progression milestones experienced by those with DMD has not been fully characterized. In 2017, Ryder et al. published a systematic review examining the epidemiology, burden, and treatment of DMD; however this review focused only on studies published between 2011 and 2015 [6]. Other reviews focused on the prevalence of DMD [13] or the impact of surgery on pulmonary decline [14]. While robust outcomes data are available from large cohort studies including the Cooperative International Neuromuscular Research Group (CINRG) [15], Duchenne Registry [16], and Centers for Disease Control and Prevention’s Muscular Dystrophy Surveillance, Tracking, and Research Network (MD STARnet) [17], a synthesis of data from recent studies is lacking [18]. The objective of this systematic review was to characterize the clinical course of DMD in the era of corticosteroid treatment in North America.

## Methods

A comprehensive search of the Medline/Medline In-Process and EMBASE databases was performed (see Additional file 1: Table S1 for search strategy), the design of which was guided by the study-specific PECOS (Population, Exposures, Comparators, Outcomes, Study design) criteria (Table 1). Studies published in English between database inception (1946) and November 2018 that reported estimates of the age at occurrence of key clinical milestones occur among males with DMD were selected. To focus on more generalizable outcomes from a more homogeneous set of patients, the review targeted observational studies from North America (or international studies including North America patients) that aimed to estimate the frequency of key clinical events from large (n > 50) samples of DMD patients treated with corticosteroids. Animal studies, or studies that included patients with other muscular dystrophies, were excluded.

**Table 1:** PECOS criteria to outline the scope of the literature review

Population	Including males with DMD in North America
Exposures/comparators	*Subgroup* Corticosteroid treatment By age By disease status at baseline
Outcomes	*Clinical/functional measures measured over a minimum of 1 year*^a^ Pulmonary function tests: Forced vital capacity, peak expiratory flow Assessment of cardiac function: Ejection fraction, left ventricular end diastolic dimension, shortening fraction *Key clinical outcomes* LOA Scoliosis Need for ventilatory support Pulmonary dysfunction Cardiac dysfunction/cardiomyopathy Mortality
Study design	Prospective or retrospective studies Case series

*DMD* Duchenne muscular dystrophy, *LOA* loss of ambulation

^a^Only commonly reported functional assessments described in included studies are listed. Other functional assessments were searched (e.g. the 6-min walk test, North Star Ambulatory Assessment, Maximum inspiratory/expiratory pressure, Forced expiratory volume) but results to include in this manuscript were not identified

Outcomes of interest that describe the clinical course of DMD included LOA, scoliosis, need for ventilatory support (stratified by any ventilation/type unspecified, non-invasive ventilation [NIV] or invasive ventilation [IV]), pulmonary dysfunction, cardiomyopathy, and mortality. Relevant measures included the mean or median age at the outcome of interest, or the percentage experiencing the outcome over time or at a particular time (*t*)*.* Scores on assessments of ambulatory, pulmonary, or cardiac function over a minimum of one year of follow-up were also included (Table 1). Two reviewers independently screened abstracts and potentially eligible full-text articles for inclusion, and any discrepancies were resolved through discussion to achieve consensus.

Data were extracted by two researchers; study characteristics extracted included authors, year, study duration, objective(s) and design, sample size, and inclusion and exclusion criteria. Patient characteristics included details of corticosteroid treatment and baseline demographics. Cohorts were classified as ‘corticosteroid-treated’ if all patients were so treated, ‘mixed corticosteroid use’ if the sample represented a mix of corticosteroid-treated and -untreated patients, and ‘likely corticosteroid-treated’ if the study was published after 2005 and did not state the sample was *untreated*. Available data on use of cardioprotective medications, such as angiotensin-converting enzyme (ACE) inhibitors, were also extracted where available.

For continuous variables, the mean, median, standard deviation (SD), confidence interval (CI), interquartile ranges (IQR), and range was extracted whenever available. For dichotomous and categorical variables, the number of patients and proportion was extracted. For studies reporting on the mean or median age at the outcome, the range of estimates was tabulated. The percentage of the sample who experienced the outcome at time of reporting was also described (where available). Data on the percentage experiencing the outcome at specific time points or over time were described using Kaplan–Meier (KM) curves, as well as presented as point estimates at time *t* by the original authors. Where available, scores on functional and clinical measures of interest over time were plotted using line graphs.

The strength of the available evidence was assessed using the STrengthening the Reporting of Observational studies in Epidemiology (STROBE) Statement for observational studies and non-randomized clinical trials [19].

## Results

**Fig. 1:**
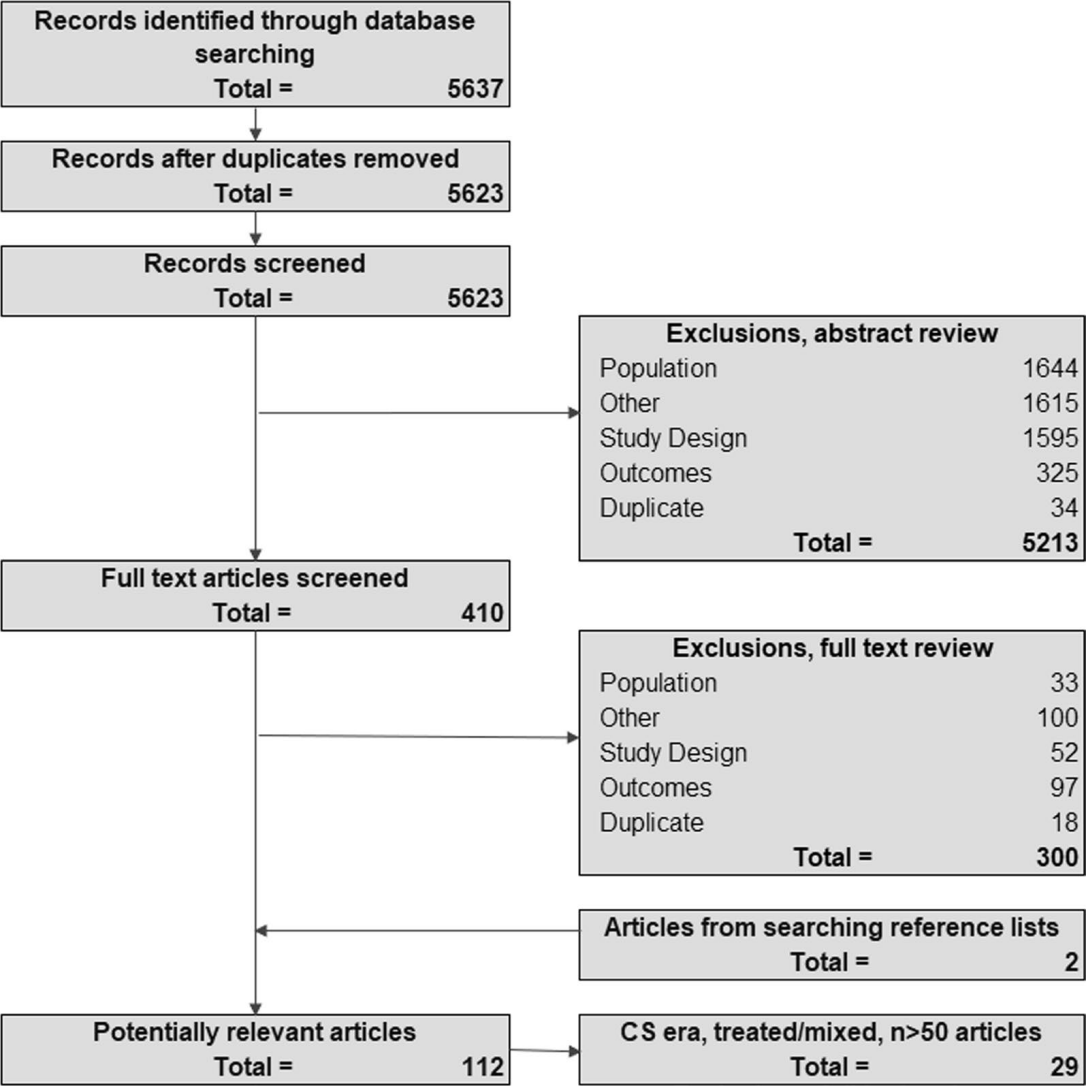
PRISMA diagram outlining study inclusion and exclusion. *PRISMA* Preferred Reporting Items for Systematic Reviews and Meta-Analyses, *CS* corticosteroid, *RTC* randomized controlled trial

The search strategy identified 5,637 potentially-relevant records; four (< 1%) were removed after de-duplication and 5,213 (92.5%) were excluded on abstract review (Fig. 1). Of the remaining 410 records, 381 were excluded on full-text review, leaving 29 eligible studies. Study designs included single-center or multicenter chart reviews and DMD registries (including 6 publications from CINRG and 4 publications from MD STARnet; Table 2). Available details of corticosteroid treatment (including the age at initiation, follow-up protocols, and frequency of reported side effects) are summarized in Additional file 1: Table S2; however, the level of detail provided varied by study, and few studies examined how variability in parameters such as age at corticosteroid initiation impacted the clinical course of DMD. Available details of treatment with cardioprotective medications are summarized in Additional file 1: Table S3. A summary of the quality of included studies in Additional file 1: Table S4.

**Table 2:** Key study and patient characteristics, included studies

Author, year	Sample characteristics	N	Geographic location	Mean age at baseline, y	Study design/data source	Study focus	Follow-up, y
Bach, 2011 + [40]	Non-ambulatory; Progressed to ventilation	134	US	19.0	Single center chart review	Survival among ventilated patients	Mean, 11.5
Bach, 2015 + [39]	Progressed to ventilation	133	US	18.6	Single center chart review	Costs and RU among ventilated patients	Mean, 8.7 Max (29)
Barber, 2013^a^ [20]	Ambulatory DMD	462	US	7.4	MD STARnet	Age at cardiomyopathy	Mean, 4
Barnard, 2018^a^ [36]	Ambulatory DMD	136	US	8.3	Multicenter chart review	qMR biomarkers in DMD	Up to 4
Bello, 2015 [28]	Ambulatory DMD	252	International^b^	6.8	CINRG-DNHS	Age at LOA and AEs of CS	Mean, 3.8
Bello, 2015 (2)^a^ [29]	Ambulatory DMD	225	International^b^	NR	CINRG-DNHS	LTBP4 and SPP1 polymorphisms on age at LOA	Mean, 4
Bello, 2016 [27]	Ambulatory DMD	157	International^b^	NR	CINRG-DNHS	Genotype x age at LOA	Mean, 4
Connolly, 2016^a^ [48]	Non-ambulatory DMD	81	US	16.8	MDA-DMD research network	Responsiveness of measures for non-ambulatory DMD	Up to 2
Deshpande, 2018^a^ [32]	Ambulatory and non-ambulatory DMD	437	US and Canada	Unclear; study entry in 2005	Administrative	Characterize clinical course; incl. in those with heart failure	Unclear; 10 per patient
Gambetta, 2018^a^ [33]	Ambulatory and non-ambulatory DMD	324	US and Canada	6.0	Multicenter chart review	Impact of genotype on outcomes	Unclear; 10 per patient
Henricson, 2017^a^ [41]	Unclear	233	International^b^	12.6	CINRG-DNHS	Impact of CS use on pulmonary function decline	Up to 9
Kim, 2015 [21]	Ambulatory DMD	220	US	Unclear; CS initiation at age 7	MD STARnet	Impact of CS on LOA	Unclear; 29
Kim, 2017 [31]	Ambulatory DMD	307	US	2.6	MD STARnet	Impact of CS on LOA	Median, 11–15
King, 2007 [22]	Ambulatory and non-ambulatory DMD	75	US	15.7	Single center chart review	Impact of CS on orthopedic outcomes	Up to 3
Labove, 2018 [23]	Cannot climb stairs	70	Canada	Unclear; age-initiated steroids 7, dx 4.2	Single center chart review	Height and age at LOA	Unclear; ≥ 7.7 per patient
Lopez-Hernandez, 2014 [24]	Unclear	432	Mexico	6.0	Multicenter chart review	Diagnosis and management of DMD in Mexico	Unclear; 20 per patient
Mayer, 2015^a^ [42]	Ambulatory and non-ambulatory	60	US	10.3	Single center chart review	Pulmonary function in DMD	Up to 5
Mcdonald, 2018 [30]	Ambulatory and non-ambulatory DMD	330	International^b^	10.7	CINRG-DNHS	Long-term effects of CS	Unclear; at > 10 per patient
Mcdonald, 2018 (2) [43]	Ambulatory and non-ambulatory	330	International^b^	11.2	CINRG-DNHS	CS use and pulmonary function in DMD	Mean, 6.1
McKane, 2017^a^ [34]	Ambulatory and non-ambulatory DMD	85	US	14.9	Single center chart review	Assoc. of body habitus with age at cardiomyopathy	Unclear; 6 per patient
Pandya, 2018^a^ [35]	Adults (non-ambulatory) with DMD	208	US	Unstated; 'adults'	MD STARnet	Clinical course among adult DMD patients	Unclear; likely > 10 per patient
Posner, 2016^a^ [25]	Ambulatory and non-ambulatory DMD	77	US	14.1	Single center chart review	Skeletal muscle and cardiac dysfunction	Unclear; 18 per patient
Schram, 2013 [45]	Boys with DMD treated with RAAS antagonists to prevent cardiomyopathy	63	Canada	9.1	Single center chart review	Characterize natural history	Mean, 11.3 (Overall)
Thomas, 2012^a^ [47]	Patients undergoing cardiac evaluation	55	US	10.6	Single center chart review	To assess elevated heart rate and cardiomyopathy onset	Mean, 4.6
Van Dorn, 2018^a^ [44]	DMD with baseline DMD with normal LV function	101	US	12.0	Multicenter chart review	Assoc. between genotype and age at LV dysfunction	Mean, 5.4
Velasco, 2007 + [38]	Non-ambulatory DMD; underwent spinal stabilization	56	US	14.0	Single center chart review	Compare rate of respiratory decline	Unclear; 12 per patient
Wang, 2018 (2) [26]	Genotyped DMD	765	US	NR	The Duchenne Registry	Age at LOA x genotype	NR
Wang, 2018^a^ [46]	DMD on cardiopulmonary therapies	57	US	18.1	Single center chart review	Progression among cardiac patients with DMD	Mean, 7.1
Wong, 2017 [37]	Early DMD; likely ambulatory and not ventilated	95	US	5.1	Single center chart review	Clinical outcomes and AEs of CS	Mean, 8.5

y = year; RU = resource use; MAX = maximum; DMD = Duchene muscular dystrophy; MD STARnet = Muscular Dystrophy Surveillance, Tracking, and Research Network, qMR = quantitative magnetic resonance; CINRG-DNHS = The Cooperative International Neuromuscular Research Group Duchene Natural History Study; LOA = loss of ambulation, AEs = adverse events; CS = corticosteroid; MDA = Muscular dystrophy association; dx = diagnosis; RAAS = Renin–angiotensin–aldosterone system; LV = left ventricular

^a^Includes samples of mixed corticosteroid treatment status, + Includes samples of unknown (but likely treated) corticosteroid treatment status

^b^The CINRG-DNHS included 63% of participants with DMD from North America (20% from Canada and 43% from the US) [15]

### Loss of ambulation

**Fig. 2:**
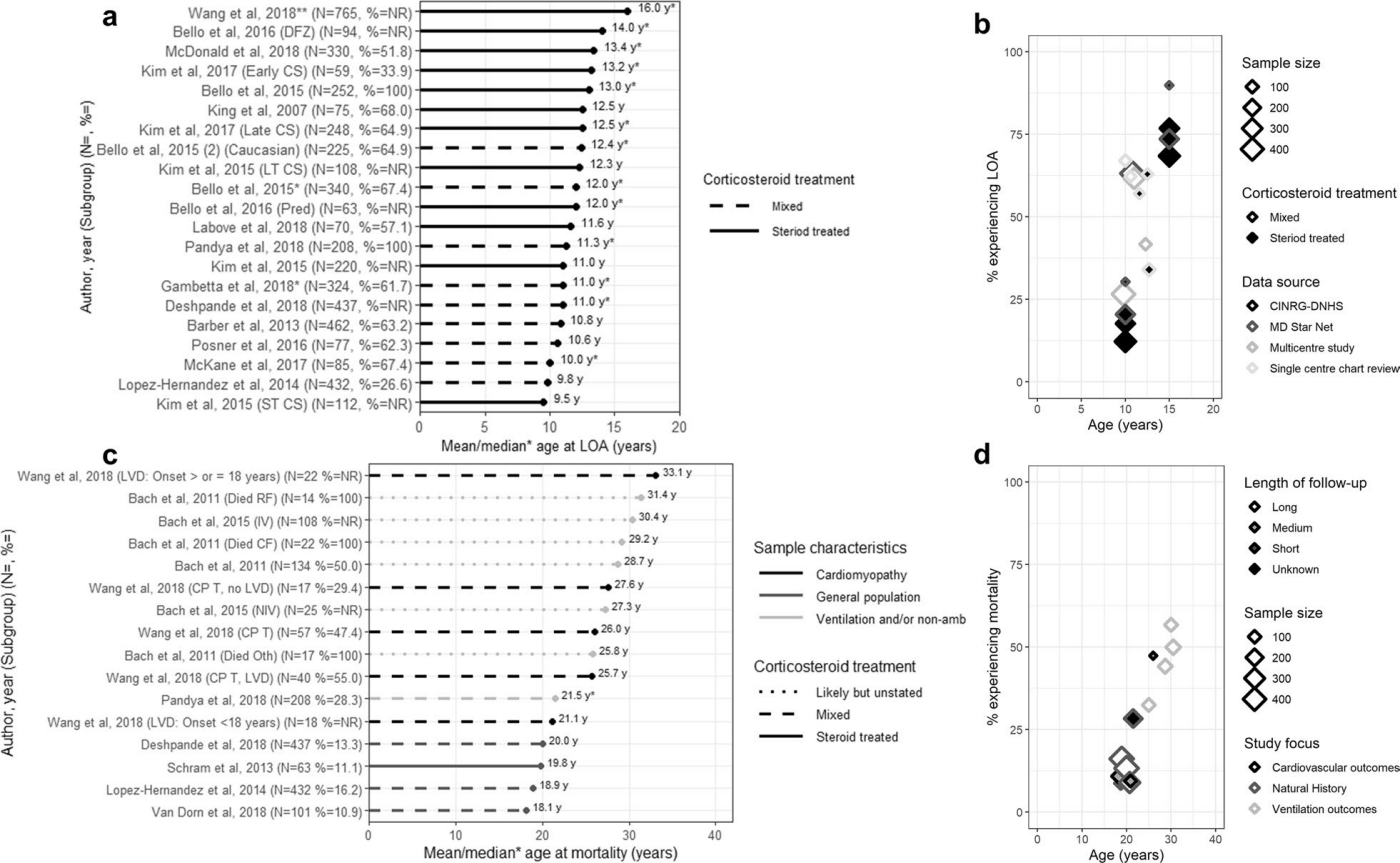
Age at LOA or mortality: **a** mean/median age at LOA; **b** LOA over time, **c** mean/median age at mortality; and **d** Mortality over time. LOA = loss of ambulation; CS = corticosteroid; LT = long-term; NR = not reported; ST = short term; yrs = years DFZ = deflazacort; NR = not reported; Pred = prednisone; yrs = years; CINRG-DNHS = The Cooperative International Neuromuscular Research Group Duchene Natural History Study; MD STARnet = Muscular Dystrophy Surveillance, Tracking, and Research Network; CM = cardiomyopathy; CPT = cardiopulmonary therapies; Died RF = died from respiratory failure; Died CF = died cardiac failure; Died Oth = died from other causes; IV = invasive ventilation; LVD = left ventricular dysfunction; NIV = non-invasive ventilation; CV = cardiovascular. *Notes* **Middle value in range of medians. Long follow up = 10–20 years; median follow up = 5.4–7.1 years; short follow up = 1.9–2 years; unknown = not reported

Six studies reported on the mean age at [20–25], 10 studies on median age at [26–35], and 13 studies on the percentage experiencing LOA (Table 2) [20, 22–25, 28–31, 33, 34, 36, 37]. Two studies provided subgroup-specific estimates [21, 31]. Among studies of corticosteroid-treated patients, the mean (SD) age at LOA ranged from 9.5 (0.2) years (among 112 patients from MD STARnet) [21] to 12.5 (3.0) years (in 68% of 75 patients from a single-center chart review [22]; Fig. 2a). Estimates were similar from the three studies reporting on mixed corticosteroid use patients; the mean ages at LOA ranged from 9.8 (2.2) years (in 26.6% of 432 Mexican DMD patients) [24] to 10.8 (2.1) years (in 63.2% of 462 patients from MD STARnet) [20]. The earliest mean age at LOA (9.5 years) was observed among patients with ≤ 3 years of corticosteroid treatment, compared with 12.3 years among those with > 3 year corticosteroid use (MD STARnet) [21].

Thirteen estimates from ten studies described median age at LOA (Fig. 2b) [26–35]. Estimates from 7 studies of corticosteroid-treated samples ranged from 12.0 (11.3–14.0) years (in 63 patients from CINRG) [29] to 16.0 (NR) years (in 765 patients from the Duchenne Registry) [26]. The latter study reported age at LOA by genotype, from 12 years (patients with exon 51 and 53 skip amenable mutations) to 20 years (patients with exon 44 skip amenable mutations). Six studies reported estimates from mixed corticosteroid use samples, and the range was tighter; from 10.0 (range: 4.0–14.0) years (in 67.4% of 85 patients from a single-center chart review) [34] to 12.4 years (in 64.9% of 225 patients from CINRG) [29].

The percentage who experienced LOA increased with time (Fig. 2c) [20, 22–25, 28–31, 33, 34, 36, 37], from 12.3% at 10 years (from 223 corticosteroid-treated CINRG patients) [30] to 89.9% at 15 years (from 53 corticosteroid-treated MD STARnet patients) [31]. Estimates from longitudinal studies report that up to 30% of DMD patients lose ambulation by 10 years (CINRG) [28], and 90% by 15 years (MD STARnet) [31]. While these effects were fairly consistent across studies of different sample sizes, mixed corticosteroid use samples tended to have higher rates of LOA at a given age than corticosteroid-treated samples.

### Scoliosis

**Fig. 3:**
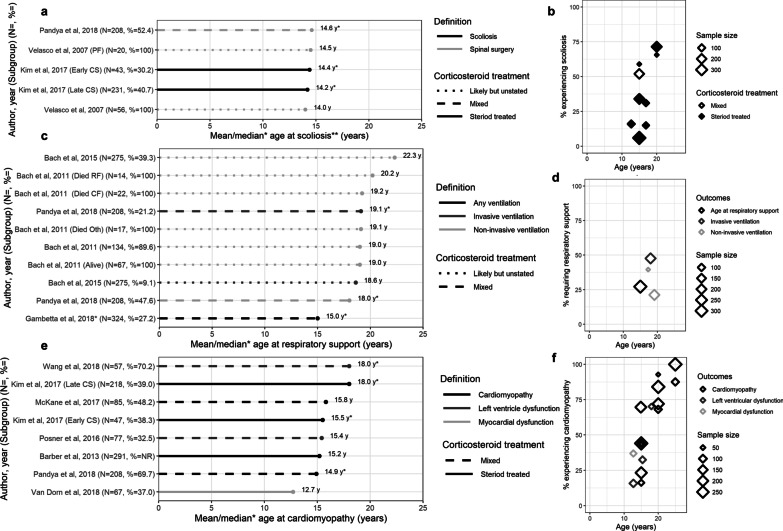
Occurrence of other key clinical milestones: **a** Mean/median age at scoliosis; **b** Percentage with scoliosis over time; **c** Mean/median age at respiratory support; **d** Percentage on respiratory support over time; **e** Mean/median age at cardiomyopathy; **f** Percentage with cardiomyopathy over time. 6MWD = 6 min walk distance; PEF = peak expiratory flow; FVC = forced vital capacity; SF = shortening fraction; LVED = left ventricular end-diastolic dimension; EF = ejection fraction. Notes: **Scoliosis includes both severe scoliosis and spinal surgery

One study reported the mean age at scoliosis [38], 2 studies the median age at scoliosis [31, 35], and 5 studies the percentage with scoliosis by age (Table 2) [22, 30, 31, 35, 37]. How scoliosis was defined varied across studies. In a single-center study of 56 patients, the mean age at spinal surgery was 14.0 years; and 14.5 years in the subset (n = 20) undergoing pulmonary function testing (Fig. 3a) [38]. The median (range) age at scoliosis surgery among a mixed corticosteroid use sample from MD STARnet was 14.6 (10.2–20.2) years (with surgery observed in 52.4% of n = 208) [35]. In the remaining study of 274 corticosteroid-treated patients (also from MD STARnet), the median (range) age (by spinal curvature > 30° *or* surgery) was 14.2 (12.5–15.6) years among the 107 patients with scoliosis [31]. The percentage with scoliosis increased with increasing age (Fig. 3b) [22, 30, 31, 35, 37]. Results from a longitudinal study from MD STARnet suggest that up to 59% of patients with DMD will have scoliosis by 15 years of age, and up to 72% by 20 years of age [31].

### Pulmonary function and need for ventilatory support

Four studies reported the mean or median age at ventilation [33, 35, 39, 40], 3 studies reported the percentage needing ventilation by age [30, 33, 35], 4 studies reported the age at transitioning to key pulmonary functional milestones [30, 41–43], and 2 studies reported pulmonary function over time (Table 2) [42, 43].

In terms of age at need for ventilation, one multicenter chart review of 324 mixed corticosteroid-treated DMD patients reported a median age at ‘any ventilation’ of 15 years (Fig. 3c) [33]. Three studies reported the age at NIV to range from a median (IQR) age of 18.0 (9.4–26.8) years (in 47.6% of 208 mixed corticosteroid-treated patients on nasal NIV from MD STARnet) [35], to a mean of 22.3 (4.7) years (in 39.3% of 275 likely-corticosteroid-treated patients receiving continuous NIV in a single-center chart review) [39]. Two studies reported age at IV; a single-center chart review reporting a mean (SD) age of 18.6 (2.3) years (in 9.1% of 275 likely-corticosteroid-treated patients with continuous tracheostomy mechanical ventilation) [39], and an MD STARnet study reporting a median (IQR) age of 19.1 (13.4–27.0) years (in 21.2% in 208 mixed-corticosteroid-treated patients with tracheostomy) [35].

The percentage of patients requiring ventilation tended to increase over time, with variability in estimates observed due to type of ventilation (Fig. 3d) [30, 33, 35]. By 20 years of age, 27.2% (n = 88) of mixed corticosteroid use patients in a multicenter chart review required ‘any ventilation’ [33]. Two studies describing NIV reported estimates of 21.2% (among 44 corticosteroid-treated patients from MD STARnet) [35], and 39.6% (among 21 mixed corticosteroid-treated patients from CINRG) [30] by 20 years. The MD STARnet study also reported that 47.6% of patients with mixed corticosteroid use were on IV by 20 years [35].

**Fig. 4:**
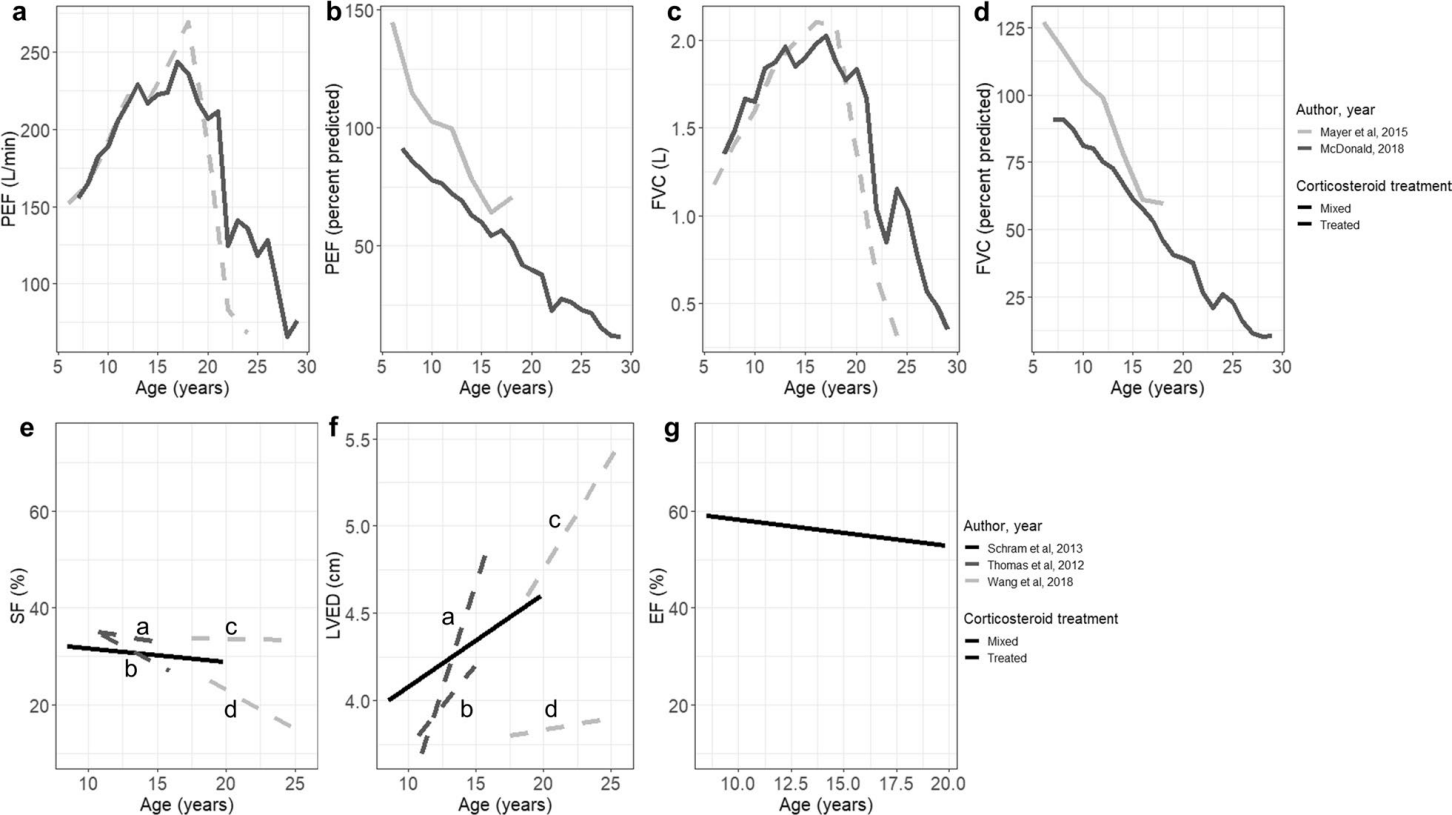
Measures of functional status over time: **a**–**d** pulmonary function measures; **e**–**g** cardiac function measures. PEF = peak expiratory flow; FVC = forced vital capacity; SF = shortening fraction; LVED = left ventricular end-diastolic dimension; EF = ejection fraction. *Notes* a = HR in the upper quartile (> 96 BPM), b = HR in the lower quartile (≤ 96 BPM), c = Left ventricular dysfunction, d = No Left ventricular dysfunction

Absolute measures of pulmonary function generally show relatively preserved function until adolescence, which declines with increasing age (Fig. 4a, c). Two studies reported absolute and percent predicted peak expiratory flow (PEF). A substantial decline in PEF was observed among 330 corticosteroid-treated CINRG patients, from 243.7 L/min (age = 17 years) to 76.1 L/min (age = 29 years). Trends were similar among 60 mixed corticosteroid-treated patients from a single-center chart review (from 269.4 L/min [age = 18 years] to 67.9 L/min [age = 24 years]) [42]. Estimates of percent predicted PEF show loss of function relative to age-matched healthy controls; the magnitude increases with age (Fig. 4a), reaching a low of 11.8% by age 29 years in the CINRG study. Those same two studies also reported FVC (L) over time (Fig. 4d) which demonstrated an initial increase in function followed by progressive decline after approximately 15 years; the percent predicted FVC showed loss of function relative to age-matched controls with increasing age, to 10.4% at 29 years of age (Fig. 4c) [42, 43].

Four studies reported the age at transitioning to key pulmonary milestones; specifically, reaching FVC < 1L, FVC < 30% or PEF < 30% [30, 41–43]. FVC < 1L was first reported at 20 years of age in the 60 mixed corticosteroid-treated patients from a single-center chart review [42] and 23 years of age in a CINRG study of 330 corticosteroid-treated patients [30]. Mean (SD) ages at FVC < 30% and PEF < 30% were similar from a CINRG study of 223 mixed corticosteroid-treated patients (FVC < 30%: 24.0 (1.5) years, and PEF < 30%: 24.9 (0.8) years); the same CINRG study also reported that 50% progressed to FVC < 30% or PEF < 30% by 25 years of age [41]. Estimates of the percentage with severe pulmonary dysfunction (FVC < 50%) at 20 years of age ranged from 13.6% [43] to 29.7% [30]. Finally, among 330 corticosteroid-treated patients from CINRG, among those with LOA at < 10 years, the median age at FVC < 1L was 18.1 years, vs 20.1 years among those with LOA between 10–13 years of age, and 24.4 years among patients with LOA at ≥ 13 years [30].

### Cardiac function and cardiomyopathy

Seven studies reported the mean or median age at diagnosis [20, 25, 26, 31, 34, 35, 44] and 9 studies reported the percentage of patients with cardiomyopathy [20, 25, 31, 34, 35, 37, 44–46]; 3 studies reported changes in cardiac function over time (Table 2) [45–47].

Of the 7 studies reporting the age at cardiomyopathy, 5 described samples *not* selected using cardiovascular-risk-related criteria (Fig. 3e) [20, 31, 34, 35, 44]. The mean (SD) age at cardiomyopathy ranged from 12.7 (3.0) years (in 37.0% of 67 corticosteroid-treated patients from a multicenter chart review) [44] to 15.8 (range: 9–29) years (in 48.2% of 85 patients of mixed corticosteroid-treatment status from a single-center chart review) [34]. Estimates of median (IQR) age at cardiomyopathy ranged from 14.9 (4.9) years (in 69.7% of 208 mixed corticosteroid-treated patients from MD STARnet) [35] to 18.0 (CI: 6.9–18.5) years (in 39.0% of 218 corticosteroid-treated patients from MD STARnet) [31]. The reported age at cardiomyopathy was lower among two studies reporting on mixed corticosteroid-treated samples either treated with cardiopulmonary therapy (median 18.0 (7.0–27.3) years, in 70.2% of 57 patients) [46], or with LV dysfunction (mean, 15.4 (8–27) years, in 32.5% of 77 patients) [25].

The percentage with cardiomyopathy was higher with increasing age (Fig. 3f) [20, 25, 31, 34, 35, 37, 44–46]; this effect was consistent across studies of different sample sizes. At 15 years of age, the percentage with cardiomyopathy ranged from 23.3% (among 218 patients who initiated corticosteroids after 5 years of age) [31] to 69.7% (among 208 mixed corticosteroid-treated patients) [35]; both estimates were from MD STARnet. By 20 years of age, the percentage with cardiomyopathy ranged from 68.2% (of 85 mixed corticosteroid-treated patients from a single-center chart review) [34] to 92.8% (of 47 patients who initiated corticosteroids before 5 years of age from MD STARnet) [31]. By age 25, the percentage with cardiomyopathy ranged from 87.6% (of 85 mixed corticosteroid-treated patients from a single-center chart review) [34] to 100% (291 corticosteroid-treated patients from MD STARnet) [20].

Measures of cardiac function show preserved function until adolescence and then decline with age (Fig. 4e–g) [45–47]. In a long-term observational study of 63 DMD patients treated with cardiopulmonary therapies and corticosteroids, the ejection fraction decreased to 53% by 20 years of age [45]. That study and two other single-center studies also reported worsening of cardiac function by left ventricular end diastolic diameter (LVED) and shortening fraction (SF) among corticosteroid-treated patients with DMD [45–47].

### Mortality

Eight studies reported the mean age [24, 32, 35, 39, 40, 44–46] and 1 study the median age at mortality [35]; 9 studies reported case fatality by age (Table 2) [24, 30, 32, 34, 35, 40, 44, 46, 48].

Of the 8 studies reporting mean (SD) age at mortality [24, 32, 35, 39, 40, 44–46], 4 were reflective of the overall DMD population [24, 32, 44, 45] one was from a sample of DMD patients with cardiomyopathy [46], and 3 were from samples of patients who were non-ambulatory or on ventilation [35, 39, 40]. From studies of the overall population, the mean (SD) age at mortality ranged from 18.1 (3.8) years (in 11% of 101 mixed corticosteroid-treated patients from a multicenter chart review) [44] to 20.0 (15–31) years (in 13% of 437 mixed corticosteroid-treated patients from an administrative database study; Fig. 2d) [32]. In the single study that described outcomes among DMD patients with cardiomyopathy, the mean (SD) age at mortality was 26.0 (6.8) years (in 47.4% of 57 mixed corticosteroid-treated patients from a single-center chart review; Fig. 2d). The mean (SD) age at mortality among DMD patients who were non-ambulatory or on ventilation ranged from 25.8 (7.8) years (in 17 likely-corticosteroid-treated patients from a single-center chart review, who died from causes other than respiratory or cardiac dysfunction) [40] to 31.4 (5.7) years (in 14 likely-corticosteroid-treated patients from that single-center chart review, with death due to respiratory complications; Fig. 2d) [40]. The median (IQR) age at mortality among DMD patients who were non-ambulatory or on ventilation was 21.5 (3.8) years (in 28.3% of 208 mixed corticosteroid-treated patients from MD STARnet; Fig. 2d) [35].

In terms of the proportion surviving over time, up to 16.2% mortality was reported by age 20 years (Fig. 2e) [24]. Estimates of survival after 20 years are available only from studies enrolling adult patients with DMD; and these reported rates of 44.2% to 56.8% mortality by age 30 years (Fig. 2e) [40].

## Discussion

A comprehensive systematic review was conducted to identify estimates of the age at key clinical milestones, and trajectories on relevant functional measures over time, among studies including North American patients with DMD. Age at LOA was the most widely reported with estimates available from many large studies; these tended to range from 10 to 14 years of age [27, 34]. However, robust data on the timing of the onset of scoliosis-, cardiac-, pulmonary- and ventilation-related outcomes were less frequently presented, particularly from large longitudinal studies. While reported estimates of the mean age at diagnosis of scoliosis were fairly consistent across studies (at 14–15 years of age), how scoliosis was classified differed widely [31, 35, 38]. Pulmonary function in DMD patients declines with age from the mid-teens [30, 41], and while most have severe pulmonary dysfunction by 25 years [30], the mean age at initiation of ventilatory support ranged from 15 to 22 years depending on the type of ventilation considered and treatment center [33, 39]. Data on age at mortality in DMD were also variable, and estimates were impacted by the inclusion criteria of the individual studies; for example estimates of mortality among those with cardiomyopathy or on ventilation were drawn from populations surviving to adulthood [40, 46]. In addition to selection criteria, factors impacting the timing of key clinical milestones include corticosteroid regimen [31] and disease genotype [26]. The findings of this review help summarize the likely timing of disease progression milestones for North American patients with DMD, and also highlight potential heterogeneity in timing observed both within and across study populations.

Estimates of time to key clinical milestones in this review included data from studies from the large North American registries (e.g. CINRG and MD STARnet), and findings are consistent with those from large observational studies and registries from outside of North America. The Translational Research in Europe—Assessment and Treatment of Neuromuscular Diseases (TREAT-NMD) network of DMD registries have published studies documenting the clinical course of patients with DMD [49–52]. In a large survey of over 1500 DMD patients that characterized the impact of corticosteroid use, mean estimates of age at LOA ranged from 10.1 (non-corticosteroid-treated patients) to 11.4 (corticosteroid-treated) years [49]. An analysis of over 5000 patients also from TREAT-NMD reported age at LOA of 13 years among corticosteroid-treated patients, and that up to 50% of patients required ventilation by 20 years of age [50]

That longitudinal data describing survival specifically among North American DMD patients are few, was one of the major gaps identified in this review. However, mortality rates from included studies were consistent with findings of two important studies on mortality in dystrophin gene-related muscular dystrophy, which did not meet the inclusion criteria for the current review as they also included patients with Becker muscular dystrophy (BMD). The first study, which was based on vital statistics, estimated that 71% of mortality among those with BMD/DMD occurred between the ages of 15 and 29 years; the authors assumed it was most likely related to DMD [53]. The second study, from MD STARnet, estimated mortality in almost 60% of that cohort by age 25 years, with most deaths occurring among those aged 20 to 25 years [54]. Further follow-up from existing large DMD cohorts will help improve contemporary estimates of the timing of key clinical milestones.

Accurately estimating the time of onset of gradually progressive manifestations of DMD can be difficult, and this along with changes in practice patterns and symptom detection, contribute to observed variability in estimates. For example, many studies reporting on scoliosis classify outcomes based on surgery, however with changing treatment patterns [55] the utility of surgery as a proxy for clinically-significant scoliosis will decrease. Similarly, recommended strategies for ventilation vary among clinical centers [39, 56, 57], and practice is changing (in particular for how IV is used) [58], which will impact the comparability of estimates of the timing of respiratory decline across studies from different periods. Finally for cardiomyopathy, with advancements in screening tools [59, 60] as well as evidence of benefits to early treatment [61], it is likely that initial signs will now be detected earlier, which would result in an apparent decrease in the mean age at cardiomyopathy over the coming years.

There are several additional factors impacting the timing of key clinical milestones that require consideration. To capture the impact of corticosteroids in the management of DMD, only studies including patients from the corticosteroid treatment era were included. While details of corticosteroid treatment regimens were extracted and reviewed, there were important limitations that precluded analyzing outcomes according to regimen. First, details on the timing of initiation, duration, type, and dose varied within and between studies. Only a small number of studies reporting on LOA presented results according to agent; but the remainder of the studies for that outcome, and all of the studies for other outcomes of interest, did not stratify by corticosteroid regimen. However, variations in corticosteroid treatment patterns (in terms of duration and dosing) may have affected the timing when patients reached LOA [28, 31, 62, 63], and other important clinical milestones [20, 31, 46, 62–65]. Evidence on the impact of early initiation of corticosteroids (e.g. before age 6 years) remains mixed [31, 66]; more work is needed to disentangle the potential confounding effect of disease severity and the potential risk for adverse effects of corticosteroid treatment on outcomes in real-world studies. Treatment with ACE inhibitors has also been shown to impact the clinical course of DMD by delaying the onset of cardiomyopathy; however, the use of ACE inhibitors remains variable [20, 67]. While it might be anticipated that studies describing later cohorts would show delayed onset of milestones that define the clinical course of DMD, the interplay between treatment advances and the impact of earlier diagnostics would make these relationships less apparent. Finally, other genetic modifiers may also play a role in the timing of DMD progression [26, 29, 50]; however, outcomes according to genotype are infrequently reported outside of treatment trials [68, 69]. The move from biomarkers to precise genetic diagnosis may also impact the apparent clinical course [70].

Variability in methodology and data sources may also have affected estimates. Data from the CINRG and MD STARnet registries, both large well-documented US cohorts that comprehensively collect longitudinal data on the clinical course of DMD, were used in ten studies within this review. Outside of those, most observational studies and treatment trials do not follow patients for a sufficient time to describe changes across the range of key clinical milestones [21, 30]. Other challenges for studying disease progression in rare diseases include small sample sizes which can amplify the impact of heterogeneity in diseases with varied clinical courses; data presented from convenience samples and case series may not be generalizable, and the impact of selection biases on outcomes (particularly for diseases with high early fatality among more severe cases) can be substantial [71, 72]. The numerous outcome measures used to assess progression in DMD also make comparisons difficult, a limitation recently acknowledged in a workshop held by the DMD research community [73]. Finally, there are useful measures for characterizing DMD progression that were infrequently reported in the studies of this review, such as the North Star Ambulatory Assessment or upper arm function, which are important in understanding patient functional status and ability to participate in activities of daily living.

Some limitations to the published data warrant mention. First, while time to event data using KM curves were presented in some studies, many reported the mean age at an occurrence where the entire sample had not experienced the event at the time of study reporting. As such, these values can be interpreted as the lower limit for when key clinical milestones will occur in DMD. Second, some measures may only be administered to individuals who still have some functional capacity (e.g. tests of ambulation), and patients unable to complete the test would have been excluded. This type of survival bias would result in an inflation of apparent functional status for cohorts as a whole. Third, mean scores on functional tests may reflect the inclusion criteria of each study, rather than the underlying distribution of scores on that functional test among the DMD population. Fourth, because of heterogeneity in designs employed, measures selected, and populations included across studies, meta-analysis was judged to be infeasible [74, 75]; as a result, overall summary estimates of the time to key clinical milestones were not calculable.

## Conclusions

This is the first systematic review of published estimates of the frequency and timing of important milestones that characterize the clinical course of DMD in the corticosteroid era. This review has also leant insight into a number of challenges in the interpretation and comparison of estimates of outcomes to characterize the clinical course of DMD. Additional studies on the ages at occurrence of other important DMD clinical milestones, and the relationships between short-term and long-term outcomes, will be valuable in the continuation of knowledge regarding disease progression in DMD.

## Supplementary Information

**Additional file 1: Supplementary Table 1**. Search strategy. **Supplementary Table 2**. Details of corticosteroid treatment, by study. **Supplementary Table 3**. Details of ACE inhibitor treatment, by study. **Supplementary Table 4**. STROBE assessments of included studies.

## Data Availability

The datasets used and/or analysed during the current study are available from the corresponding author on reasonable request.

## Abbreviations

ACE: Angiotensin-converting enzyme
BMD: Becker muscular dystrophy
CI: Confidence interval
CINRG: Cooperative International Neuromuscular Research Group
DMD: Duchenne muscular dystrophy
FVC: Forced vital capacity
IQR: Interquartile ranges
IV: Invasive ventilation
KM: Kaplan–Meier
LOA: Loss of ambulation
LV: Left ventricle
LVED: Left ventricular end diastolic diameter
MD STARNet: Muscular Dystrophy Surveillance, Tracking, and Research Network
NIV: Non-invasive ventilation
PEF: Peak expiratory flow
SD: Standard deviation
SF: Shortening fraction
STROBE: STrengthening the Reporting of Observational studies in Epidemiology
TREAT-NMD: Translational Research in Europe—Assessment and Treatment of Neuromuscular Diseases
