# Supplementary material for: The clinical course of Duchenne muscular dystrophy in the corticosteroid treatment era: a systematic literature review
Source: Orphanet J Rare Dis. 2021 May 22;16:237. doi: 10.1186/s13023-021-01862-w (PMC8141220; doi:10.1186/s13023-021-01862-w)
Supplement: Supplementary file 1 — Additional file 1: Supplementary Table 1. Search strategy. Supplementary Table 2. Details of corticosteroid treatment, by study. Supplementary Table 3. Details of ACE inhibitor treatment, by study. Supplementary Table 4. STROBE assessments of included studies. [file 13023_2021_1862_MOESM1_ESM.docx]

**Supplementary tables**

**Supplementary table 1 Search strategy**

|  | | **Search term** | **Results** |
| --- | --- | --- | --- |
| Database: | Embase 1974-November 19, 2018 | | |
|  | Ovid MEDLINE(R) and Epub Ahead of Print, In-Process & Other Non-Indexed Citations, Daily and Versions(R) 1946-November 12, 2018 | | |
| Date: | November 20, 2018 | | |
|  | 1 | DMD.ti,ab,ot. | 16483 |
|  | 2 | (duchenne$ adj3 (syndrome$ or morbus)).ti,ab,ot,hw. | 141 |
|  | 3 | ((duchenne$ or pseudo hypertrophic or pseudohypertrophic) adj3 dystroph$).ti,ab,ot,hw. | 28101 |
|  | 4 | Duchenne muscular dystrophy/ | 19221 |
|  | 5 | or/1-4 | 31856 |
|  | 6 | animal/ | 7685785 |
|  | 7 | animal experiment/ | 2292060 |
|  | 8 | (rat or rats or mouse or mice or murine or rodent or rodents or hamster or hamsters or pig or pigs or porcine or rabbit or rabbits or animal or animals or dogs or dog or cats or cow or bovine or sheep or ovine or monkey or monkeys).ti,ab,ot,hw. | 13125181 |
|  | 9 | or/6-8 | 13125181 |
|  | 10 | exp human/ | 36435749 |
|  | 11 | human experiment/ | 424404 |
|  | 12 | or/10-11 | 36437157 |
|  | 13 | 9 not (9 and 12) | 9690528 |
|  | 14 | 5 not 13 | 25969 |
|  | 15 | exp history/ or natural history.mp. | 744483 |
|  | 16 | disease course/ or (disease course or disease progression).mp. | 681270 |
|  | 17 | exp mortality/ or mortality.mp. | 2418732 |
|  | 18 | exp death/ or death.mp. | 2042037 |
|  | 19 | exp intelligence/ or intelligence.mp. | 242732 |
|  | 20 | exp intelligence tests/ or intelligence test.mp. | 35301 |
|  | 21 | exp cognition/ or (cognition or cogniti* test or cogniti* assessment).mp. | 2230239 |
|  | 22 | exp wheelchairs/ or wheelchair.mp. | 18897 |
|  | 23 | exp walking/ or (mobili#ation or ambulat* or walking).mp. | 671189 |
|  | 24 | exp respiratory function tests/ or (respiratory function test or respiratory failure or lung function or pulmonary dysfunction).mp. | 560595 |
|  | 25 | exp respiratory insufficiency/ or respiratory insufficiency.mp. | 152563 |
|  | 26 | exp cardiomyopathies/ or cardiomyopath*.mp. | 259990 |
|  | 27 | exp respiration/ or (breathing or respirat* or ventilat*).mp. | 1936473 |
|  | 28 | exp scoliosis/ or scoliosis.mp. | 55485 |
|  | 29 | or/15-28 | 9942774 |
|  | 30 | 14 and 29 | 8926 |
| Embase | 31 | 30 use oemezd | 6080 |
|  | 32 | DMD.ti,ab,ot. | 16483 |
|  | 33 | (duchenne$ adj3 (syndrome$ or morbus)).ti,ab,ot,hw. | 141 |
|  | 34 | ((duchenne$ or pseudo hypertrophic or pseudohypertrophic) adj3 dystroph$).ti,ab,ot,hw. | 28101 |
|  | 35 | Muscular Dystrophy, Duchenne/ | 12575 |
|  | 36 | or/32-35 | 31856 |
|  | 37 | exp animals/ not (exp animals/ and humans/) | 16127696 |
|  | 38 | 36 not 37 | 20562 |
|  | 39 | exp Natural History/ or natural history.mp. | 367426 |
|  | 40 | exp Disease Progression/ or disease progression.mp. | 395016 |
|  | 41 | exp mortality/ or mortality.mp. | 2418732 |
|  | 42 | exp mortality rate/ or mortality rate.mp. | 539456 |
|  | 43 | exp mortality risk/ or mortality risk.mp. | 45409 |
|  | 44 | exp death/ or death.mp. | 2042037 |
|  | 45 | exp intelligence/ or (intelligence or intelligence test).mp. | 242732 |
|  | 46 | exp cognition/ or cognition.mp. | 2224544 |
|  | 47 | exp wheelchair/ or wheelchair.mp. | 18897 |
|  | 48 | exp mobilization/ or (mobili#ation or ambulat*).mp. | 458515 |
|  | 49 | exp lung function/ or (lung function or pulmonary dysfunction).mp. | 199860 |
|  | 50 | exp respiratory failure/ or (respiratory failure or respiratory function test).mp. | 175158 |
|  | 51 | exp respiratory insufficiency/ or respiratory insufficiency.mp. | 152563 |
|  | 52 | exp cardiomyopathy/ or cardiomyopath*.mp. | 259990 |
|  | 53 | exp breathing/ or (breathing or respirat* or ventilat*).mp. | 1936473 |
|  | 54 | exp scoliosis/ or scoliosis.mp. | 55485 |
|  | 55 | exp cognition assessment/ or (cogniti* assessment or cogniti* test).mp. | 38927 |
|  | 56 | or/39-55 | 9087100 |
|  | 57 | 38 and 56 | 5501 |
| Medline | 58 | 57 use ppezv | 2683 |
|  | 59 | limit 31 to english | 5614 |
|  | 60 | limit 58 to English | 2403 |
|  | 61 | remove duplicates from 59 | 5512 |
|  | 62 | remove duplicates from 60 | 2392 |
|  | 63 | 61 and 62 | 0 |
|  | 64 | 61 or 62 | 7904 |
|  | 65 | letter/ | 1999659 |
|  | 66 | editorial/ | 1070332 |
|  | 67 | comment/ | 741052 |
|  | 68 | case report.tw. | 646976 |
|  | 69 | or/65-68 | 3894577 |
|  | 70 | 64 not 69 | 7379 |
|  | 71 | limit 70 to yr="1946 -2005" | 1916 |
|  | 72 | limit 70 to yr="2006-current" | 5453 |
|  | 73 | remove duplicates from 71 | 1310 |
|  | 74 | remove duplicates from 72 | 4327 |
|  | 75 | 73 or 74 | **5637** |

**Supplementary table 2 Details of corticosteroid treatment, by study**

| **Citation** | **n treated** | **Treatment** | **Dose** | **Frequency** | **Mean (SD) tx duration** | **Mean age at initiation, y** | **Follow-up protocols** | **Adverse events reported** | **Impact of age at initiation on key outcomes** |
| --- | --- | --- | --- | --- | --- | --- | --- | --- | --- |
| Bach, 2011+ | Unknown | | | | | | | | |
| Bach, 2015+ | Unknown | | | | | | | | |
| Barber, 2013* | 291 | Pred, PRDL, or DFZ | -- | -- | 4.1 (3.4) y | 7.4 (2.5) | -- | -- | -- |
| Barnard, 2018* | 99 | No further details provided | | | | | | | |
| Bello, 2015 | 94 | PRED | 75† | qd | -- | 6.6 (1.9) | -- | Weight gain (67%); Cushingoid (50%); behavior change (30%); growth delay (27%); cataracts (5%); low BMD/fx (22%); skin (11%) | -- |
|  | 80 | DFZ | 83† | qd | -- | 7.2 (2) | -- | Weight gain (63%); Cushingoid (72%); behavior change (33%); growth delay (60%); cataracts (29%); low BMD/fx (25%); skin (8%) |  |
|  | 23 | PRED | 94† | -- | -- | 7 (2) | -- | Weight gain (70%); Cushingoid (48%); behavior change (52%); growth delay (17%); cataracts (4%); low BMD/fx (9%); skin (13%) |  |
|  | 21 | PRED to DFZ | 71† | qd | -- | 6.2 (2.3) | -- | Weight gain (76%); Cushingoid (62%); behavior change (52%); growth delay (57%); cataracts (14%); low BMD/fx (24%); skin (24%) |  |
|  | 19 | PRED | 131† | High-dose q2w | -- | 7 (2.1) | -- | Weight gain (79%); Cushingoid (37%); behavior change (42%); growth delay (5%); cataracts (11%); low BMD/fx (26%); skin (37%) |  |
|  | 15 | PRED to DFZ | 85† | -- | -- | 5.2 (1.5) | -- | Weight gain (80%); Cushingoid (67%); behavior change (73%); growth delay (53%); cataracts (40%); low BMD/fx (40%); skin (27%) |  |
|  | 8 | DFZ | 82† | -- | -- | 6.2 (1.7) | -- | Weight gain (38%); Cushingoid (25%); behavior change (50%); growth delay (25%); cataracts (0%); low BMD/fx (0%); skin (0%) |  |
|  | 5 | PRED | 71† | q5w | -- | 8 (1.1) | -- | Weight gain (23%); Cushingoid (8%); behavior change (15%); growth delay (8%); cataracts (0%); low BMD/fx (23%); skin (8%) |  |
|  | 4 | PRED | 38† | Every other day | -- | 9.1 (1.9) | -- |  |  |
|  | 2 | PRED | 47† | 10 d on/off | -- | 9.4 (0.4) | -- |  |  |
|  | 2 | PRED | 50† | 10 d/mo | -- | 6.1 (0.4) | -- |  |  |
|  | 2 | DFZ | 136† | High-dose q2w | -- | 11.5 (2.9) | -- | -- |  |
|  | 1 | DFZ | 65† | Every other day | -- | 3.6 (0) | -- | -- |  |
|  | 1 | PRED | 48† | 2qd | -- | 6.9 (0) | -- | -- |  |
| Bello, 2015 (2)* | 252 | No further details provided | | | | | | | |
| Bello, 2016 | 63 | Pred or PRDL | 0.56 mg/kg | qd | -- | -- | -- | -- | -- |
|  | 94 | DFZ | 0.75 mg/kg | qd | -- | -- | -- | -- | -- |
| Connolly, 2016* | 19 | NR | -- | q2w | -- | -- | -- | -- | -- |
|  | 25 | NR | -- | qd | -- | -- | -- | -- | -- |
| Deshpande, 2018* | 336 | No further details provided | | | | | | | |
| Gambetta, 2018* | 199 | NR | -- | -- | -- | 6 (5-8)** | -- | -- | -- |
| Henricson, 2017* | 186 | No further details provided | | | | | | | |
| Kim, 2015 | 141 | Overall (≥3mo) Pred | -- | -- | 3.1 (0.2) y | 7.1 (0.1) | -- | -- | -- |
|  | 49 | Overall (≥3mo) DFZ | -- | -- | 3.6 (0.3) y | 6.8 (0.2) | -- | -- | -- |
|  | 30 | Overall (≥3mo) Pred + DFZ | -- | -- | 4.2 (0.4) y | 7.0 (0.2) | -- | -- | -- |
| Kim, 2017 | 67 | Early CS-treated (≤5 years) Pred or PRDL or DFZ | -- | -- | ≥6mo | 4.2 | -- | Fx (40%) | Early-CS tx associated with risk of earlier CM (HR, 2.0 [1.2, 3.4]) and fx (HR, 2.3 [1.4, 3.7]). Early CS-tx also associated with slightly decreased respiratory function. Age at LOA, scoliosis did not differ statistically by tx. |
|  | 316 | Late CS-treated (>5 years) Pred or PRDL or DFZ | -- | -- | ≥6mo | 7.6 | -- | Fx (35%) |  |
| King, 2007 | 36 | Pred | 0.75 mg/kg | qam | 8.04 y | -- | Calcium (carbonate 350 mg tid or with vit D [750 to 1,200 mg] qd); dietary counseling; BMD by DEXA every 6 mo to 2 y. | Vertebral fx (32%); long bone fx (39%);  51% occurred while ambulatory | -- |
|  | 25 | DFZ | 0.9 mg/kg | qam | 8.04 y | -- |  |  |  |
|  | 14 | Pred + DFZ | -- | qam | 8.04 y | -- |  |  |  |
| Labove, 2018 | 70 | NR |  |  |  | 7.5** | -- | Growth delay: At 10y, boys with LOA after 11.6 y were 11.9 cm shorter than boys with LOA before 11.6 y. |  |
| Lopez-Hernandez, 2014 | 96 | No further details provided | | | | | | | |
| Mayer, 2015* | 16 | Pred or PRDL | 0.3 mg/kg | qd | -- | -- | -- | -- | -- |
|  | 11 | DFZ | 0.4 mg/kg | qd | -- | -- | -- | -- | -- |
| McDonald, 2018 | 40 | Pred or PRDL | -- | qd | 1729 (1371) days | -- | -- | Weight gain (14%); Cushingoid appearance (9%); behavior changes (6%); growth delay (4%); fx (3%); cataracts (<1%) | -- |
|  | 107 | DFZ | -- | qd | 2922 (2133) days | -- | -- | Weight gain (5%); Cushingoid appearance (6%); behavior changes (3%); growth delay (5%); fx (1%); cataracts (3%) |  |
|  | 63 | Pred or PRDL | -- | Alternatingꝉ | 2276 (1605) days | -- | -- | -- |  |
|  | 50 | Pred or PRDL or DFZ (switched) | -- | -- | 3239 (1760) days | -- | -- | -- |  |
|  | 84 | All other CS tx | -- | -- | 2317 (1652) days | -- | -- | -- |  |
| McDonald, 2018 (2) | 40 | Pred or PRDL | -- | qd | 1729 (1371) days | -- | -- | -- | -- |
|  | 107 | DFZ | -- | qd | 2922 (2133) days | -- | -- | -- | -- |
|  | 63 | Pred or PRDL | -- | Alternatingꝉ | 2276 (1605) days | -- | -- | -- | -- |
|  | 50 | Pred or PRDL or DFZ (switched) | -- | -- | 3239 (1760) days | -- | -- | -- | -- |
|  | 84 | All other CS tx | -- | -- | 2317 (1652) days | -- | -- | -- | -- |
| McKane, 2017* | 44 | -- | -- | -- | 4** | -- | -- | Weight gain | -- |
| Pandya, 2018* | 101 | CS (any) | -- | -- | 4.9 (11.1) y** | 7.6 (2.4) | -- | -- | -- |
|  | 77 | CS (>6 months) | -- | -- | 4.9 (11.1) y** | 7.6 (2.4) | -- | -- | -- |
| Posner, 2016* | 60 | CS (overall) | -- | -- | 3.4 (2.5) y | -- | -- | -- | -- |
|  | 35 | CS (non-ambulatory) | -- | -- | 3.6 (2.8) y | -- | -- | -- | -- |
|  | 25 | CS (ambulatory) | -- | -- | 3.2 (2.1) y | -- | -- | -- | -- |
| Schram, 2013 | 63 | Pred or DFZ | 0.5-0.7 or 0.9 mg/kg | qd | 11 (4.8) y | 8.6 (3.5) | Calcium (250 mg tid), vit D (400 IU daily); cardiology assessment; cardioprotective meds as needed. | Patients on CS shorter than those not (149 ± 14 cm vs. 167 ± 11 cm, p < 0.0001); significantly lower BMI (19 ± 7 kg/m2 vs. 24 ± 6 kg/m2, p = 0.0017). | -- |
| Thomas, 2012* | 19 | NR | -- | -- | >12 mos | -- | -- | -- | -- |
| Van Dorn, 2018* | 67 | Pred or DFZ; stable CS >1 y prior to entry | -- | -- | ≥1 y | -- | -- | -- | -- |
| Velasco, 2007+ | Unknown | | | | | | | | |
| Wang et al, 2018* | 15 | Pred and other | -- | -- | -- | -- | -- | -- | -- |
|  | 6 | Pred and other (no LVD) | -- | -- | -- | -- | -- | -- | -- |
|  | 9 | Pred and other (with LVD) | -- | -- | -- | -- | -- | -- | -- |
|  | 10 | Pred and other | -- | -- | >2 yrs | -- | -- | -- | -- |
|  | 4 | Pred (no LVD) | 20 mg | qd | >2 yrs | -- | -- | -- | -- |
|  | 6 | Pred (with LVD) | 20 mg | qd | >2 yrs | -- | -- | -- | -- |
| Wang et al, 2018 (2) | 765 | No further details provided | | | | | | | |
| Wong et al, 2017 | 86 | DFZ | 0.9 mg/kg | qd | 8.5 (1.6) y | 5.1 (1.3) | Vit D supplement; bisphosphonates or growth hormone as needed; vit D insufficiency or deficiency treated; behavioral changes or exacerbation of difficulties noted. | Facial fullness (19.6% mild; 48.5% moderate), Cushingoid (2.1%). n=1 stopped CS for behavioral concerns, short stature. n=4 treated for severe refractory weight gain (after optimizing diet, CS type and dose). | -- |
|  | 11 | Pred | 0.75 mg/kg | qd | 8.5 (1.6) y | 5.1 (1.3) |  |  |  |
|  | 59 | Pred or DFZ (10-<13y) | 0.75 or 0.9 mg/kg | qd | 7.8 (1.4) y | 4.9 (1.3) |  | Lower weight percentile vs previous (84.2%); decreased BMD (lumbar spine, 35.6%; distal femur; 62.1%); asymptomatic spine compression (74.6%); long bone fx (23.7%); cataracts (11.9%) |  |
|  | 38 | Pred or DFZ (13-<16y) | 0.75 or 0.9 mg/kg | qd | 9.5 (1.3) y | 5.3 (1.3) |  | Same/lower weight percentile vs. previous (89.5%); decreased BMD (lumbar spine, 40.5%; distal femur; 86.8%); spine compression deformities (78.9%); long bone fx (39.5%); cataracts (28.9%) |  |

Abbreviations: BMD = bone mineral density; BMI = body mass index; CI = confidence interval; CS = corticosteroid; CM = cardiomyopathy; DFZ = deflazacort; fx = fracture; HR = hazard ratio; mo = months; NR = not reported; Pred = prednisone, PRDL = prednisolone; qd = once daily; qam = daily before noon qxw = x times/week; SD = standard deviation; tx = treatment; tid = three times a day; vit = vitamin; y = year.

Notes: * Includes samples of mixed corticosteroid treatment status, ** median, + Includes samples of unknown (but likely treated) corticosteroid treatment status, † Dose is indicated as % of standard mg/kg/d (0.75 mg/kg for PRED or 0.9 mg/kg for DFZ as applicable).

**Supplementary table 3 Details of ACE inhibitor treatment, by study**

| **Author, year** | **N** | **% treated with ACE inhibitor** | **Median age at ACE inhibitor initiation, y** |
| --- | --- | --- | --- |
| Bach, 2011+(41) | 134 | *--* | *--* |
| Bach, 2015+(40) | 133 | *--* | *--* |
| Barber, 2013*(21) | 462 | <8.0 prior to CM onset | *--* |
| Barnard, 2018*(37) | 136 | *--* | *--* |
| Bello, 2015(29) | 252 | *--* | *--* |
| Bello, 2015 (2)*(30) | 225 | *--* | *--* |
| Bello, 2016(28) | 157 | *--* | *--* |
| Connolly, 2016*(49) | 81 | *--* | *--* |
| Deshpande, 2018*(33) | 437 | *--* | *--* |
| Gambetta, 2018*(34) | 324 | *--* | *--* |
| Henricson, 2017*(42) | 233 | *--* | *--* |
| Kim, 2015(22) | 220 | *--* | *--* |
| Kim, 2017(32) | 307 | *--* | *--* |
| King, 2007(23) | 75 | *--* | *--* |
| Labove, 2018(24) | 70 | *--* | *--* |
| Lopez-Hernandez, 2014(25) | 432 | *--* | *--* |
| Mayer, 2015*(43) | 60 | *--* | *--* |
| McDonald, 2018(31) | 330 | *--* | *--* |
| McDonald, 2018 (2)(44) | 330 | *--* | *--* |
| McKane, 2017*(35) | 85 | *--* | *--* |
| Pandya, 2018*(36) | 208 | 75.4† | 19.0 (7.0 to 27.0)† |
| Posner, 2016*(26) | 77 | *--* | *--* |
| Schram, 2013(46) | 63 | 86.0 | *--* |
| Thomas, 2012*(48) | 55 | *--* | *--* |
| Van Dorn, 2018*(45) | 101 | *--* | *--* |
| Velasco, 2007+(39) | 56 | *--* | *--* |
| Wang, 2018 (2)(27) | 765 | *--* | *--* |
| Wang, 2018*(47) | 57 | 89.5‡ | *--* |
| Wong, 2017(38) | 95 | *--* | *--* |

Abbreviations: ACE = Angiotensin-converting enzyme; y = year.

Notes: * Includes samples of mixed corticosteroid treatment status, + Includes samples of unknown (but likely treated) corticosteroid treatment status; † ACE inhibitor or beta-blockers, ‡ ACE inhibitor or Angiotensin receptor blockers

**Supplementary table 4 STROBE assessments of included studies**

| **Author, year** | **Strobe items** | | | | | | | | | | |
| --- | --- | --- | --- | --- | --- | --- | --- | --- | --- | --- | --- |
|  | **A** | **B** | **C** | **D** | **E** | **F** | **G** | **H** | **I** | **J** | **K** |
| Bach, 2011+ | Y | Y | Y | Y | Y | Y | Y | Y | Y | N | Y |
| Bach, 2015+ | Y | Y | Y | Y | Y | Y | N | Y | Y | N | Y |
| Barber, 2013* | Y | Y | Y | Y | Y | Y | Y | Y | Y | N | Y |
| Barnard, 2018* | Y | Y | Y | Y | Y | Y | Y | Y | Y | Y | Y |
| Bello, 2015 | Y | Y | Y | Y | Y | Y | Y | Y | Y | N | Y |
| Bello, 2015 (2)* | Y | Y | Y | Y | Y | Y | Y | Y | Y | N | Y |
| Bello, 2016 | Y | Y | Y | Y | Y | Y | Y | Y | Y | N | Y |
| Connolly, 2016* | Y | Y | Y | Y | Y | Y | Y | Y | Y | Y | Y |
| Deshpande, 2018* | Y | Y | Y | N | N | N | N | Y | Y | N | N |
| Gambetta, 2018* | Y | Y | Y | Y | N | N | N | Y | Y | N | N |
| Henricson, 2017* | Y | Y | Y | P | P | P | N | Y | Y | N | P |
| Kim, 2015 | Y | Y | Y | Y | Y | Y | Y | Y | Y | N | Y |
| Kim, 2017 | Y | Y | Y | Y | Y | Y | Y | Y | Y | N | Y |
| King, 2007 | Y | Y | Y | Y | Y | Y | Y | Y | Y | N | Y |
| Labove, 2018 | Y | P | Y | N | N | N | N | Y | Y | N | P |
| Lopez-Hernandez, 2014 | Y | Y | Y | Y | Y | Y | Y | Y | Y | N | Y |
| Mayer, 2015* | Y | Y | Y | Y | Y | Y | Y | Y | Y | N | Y |
| McDonald, 2018 | Y | Y | Y | Y | Y | Y | Y | Y | Y | N | Y |
| McDonald, 2018 (2) | Y | Y | Y | Y | Y | Y | Y | Y | Y | Y | Y |
| McKane, 2017* | Y | Y | Y | Y | Y | Y | Y | Y | Y | N | Y |
| Pandya, 2018* | Y | Y | Y | P | P | Y | Y | Y | Y | N | Y |
| Posner, 2016* | Y | Y | Y | Y | Y | Y | Y | Y | Y | N | Y |
| Schram, 2013 | Y | Y | Y | Y | Y | Y | Y | Y | Y | N | Y |
| Thomas, 2012* | Y | Y | Y | Y | Y | Y | Y | Y | Y | N | Y |
| Van Dorn, 2018* | Y | Y | Y | Y | Y | Y | Y | Y | Y | N | Y |
| Velasco, 2007+ | Y | Y | Y | Y | Y | Y | Y | Y | Y | N | Y |
| Wang, 2018 (2) | Y | Y | Y | Y | Y | Y | Y | Y | Y | N | Y |
| Wang, 2018* | Y | Y | Y | Y | Y | Y | Y | Y | Y | N | Y |
| Wong, 2017 | Y | Y | Y | Y | Y | Y | Y | Y | Y | N | Y |

A, Objectives and prespecified hypothesis in the introduction; B, Present key elements of study design early in paper, including setting; C, Eligibility criteria of cohort in methods; D, Define all outcomes and methods of assessment; E, Potential biases addressed; F, Study size calculated; G, Statistical methods described; H, Report characteristics of study participants; I, Report outcomes and exposures; J, Mention of how missing data was handled; K, Limitations of the study and the generalizations mentioned; Y, Yes; N, No; P, Partially.

Notes: * Includes samples of mixed corticosteroid treatment status, + Includes samples of unknown (but likely treated) corticosteroid treatment status.ss
